# Is nebulized saline a placebo in COPD?

**DOI:** 10.1186/1471-2466-4-9

**Published:** 2004-09-30

**Authors:** Shahina Y Khan, B Ronan O'Driscoll

**Affiliations:** 1 Dept of Respiratory Medicine, Hope Hospital, Salford, M6 8HD UK

## Abstract

**Background:**

Many trials of nebulized therapy have used nebulized saline as a "placebo". However, nebulized isotonic saline is sometimes used to assist sputum expectoration and relieve breathlessness in COPD patients. We designed this study to establish if nebulized saline had a placebo effect or a clinical effect.

**Methods:**

40 patients were studied following hospital admission for exacerbated COPD (mean FEV1 30% predicted). Patients were randomised to single-blind administration of either 4 mls of nebulized isotonic saline using an efficient nebulizer (active group n = 20) or an inefficient nebulizer (placebo group n = 20). Spirometry and subjective breathlessness scores (Modified Likert Scale) were measured before nebulized treatment and 10 minutes after treatment.

**Results:**

There was no significant change in FEV1 after active or placebo nebulized saline treatment. Patients reported a 4% improvement in mean breathlessness score following placebo (Wilcoxon test; p = 0.37) compared with 23% improvement following active nebulized saline (p = 0.0001). 65% of patients given active nebulized saline but only 5% of the placebo group reported that mucus expectoration was easier after the treatment.

**Conclusions:**

This study lends support to the current use of nebulized saline to relieve breathlessness (possibly by facilitating sputum clearance) in COPD patients. Lung function was not affected. Nebulized saline can therefore be used as a placebo in bronchodilator studies involving COPD patients but it cannot be used as a placebo in trials assessing symptom relief.

## Background

Nebulized saline is used by some doctors and physiotherapists to assist mucus clearance and to relieve breathlessness in patients with COPD, bronchiectasis and Cystic Fibrosis. This practice is justified by a small number of studies which have demonstrated enhanced sputum expectoration or improved breathlessness after nebulized saline or humidified oxygen [1-4]. Nebulized hypertonic or isotonic saline has been used to obtain induced sputum specimens from patients with asthma and COPD for diagnostic and experimental purposes [5-9]. For example, Vlachos-Mayer and colleagues [5] used increasing strengths of nebulized saline (from isotonic up to 5%) to induce sputum in 304 patients with asthma and 25 patients with COPD. Satisfactory specimens were obtained in 93% of cases, 17% of asthmatic patients and 56% of COPD patients required only isotonic saline to achieve sputum induction.

However, nebulized saline has also been used as a placebo in several trials involving nebulized bronchodilator therapy [10,11]. For example, Jenkins et al [10] found in a double blind study that patients reported clinical benefit from nebulized saline (with MDI bronchodilator therapy) which was similar to the subjective response to nebulized bronchodilator therapy given with placebo MDI therapy. It was assumed that these patients had a placebo response to nebulized saline but it is also possible that they may have experienced a non-bronchodilator benefit from nebulized saline. We have designed a trial to determine whether the symptomatic benefit associated with nebulized saline use in clinical trials is a placebo effect or a non-bronchodilator therapeutic effect.

## Methods

40 patients were studied during a hospital admission for an exacerbation of COPD. Patients were recruited at a time when their condition had stabilized prior to their planned discharge from hospital. Clinical details of the patients are summarised in table 1. Six patients undertook both limbs of the study (partial crossover design).

**Table 1:** Patient Characteristics

	**Active group**	**Placebo group**
**Gender**	13 Male, 7 Female	12 Male, 8 Female
**Age (Mean, SD)**	68.1 +/- 7.2 Range 54–79	67.3 +/- 7.4 Range 58–79
**Mean FEV1** **95% CI**	0.77 Litres 0.65–0.89	0.78 Litres 0.61–0.95
**FEV1 as % predicted (Mean and SD)**	29.9 (10.0)	30.6 (14.8)

Patients were randomised to receive 4 mls of 0.9% saline using an efficient nebulizer system (active group) or an inefficient nebulizer system (placebo group). The active nebulizer was a System 22 Acorn nebulizer (Medic-Aid, Bognor Regis UK Ltd) driven by the hospital's piped oxygen supply at a flow rate of 9 l/min for 10 minutes. This nebulizer system was found to deliver 95% of particles in the size range 2.5 to 2.8 microns using a Malvern laser system. (Measurement courtesy of Dr Steve Newman, Principal Physicist, Royal Free Hospital, London, UK). This small particle size was selected to achieve effective delivery to the airways. The placebo nebulizer was an old model (1980s) Bard Inspiron nebulizer (no longer manufactured) driven by oxygen at a flow rate of 3 l/min. This nebulizer system delivered 95% of particles in the size range 9.5 to 9.9 microns. This particle size was selected to achieve a placebo effect with deposition in the tubing of the system and in the pharynx but little penetration to the airways [12]. Both nebulized treatments were administered by mouthpiece to avoid nasal deposition of saline droplets and to make it less likely that patients would notice that the output from the placebo system was different to previous nebulized treatment which they had received.

The trial was conducted in a single-blind manner. 40 slips of paper were labelled "Treatment A" or "Treatment B" and placed in opaque brown envelopes. These were shuffled in random order and each patient was asked to select one envelope. This was then opened by the investigator and the appropriate treatment was administered (A active, B Placebo). For the six patients who took part in the study twice, the second treatment consisted of whichever treatment they had not received previously.

Patients were told that we wished "to observe the effects of a nebulized treatment which is not a new or experimental drug". They were not informed of the exact nature of the nebulized treatments as this might have led patients to try to guess if the treatment which they received was a "placebo". The Ethics Committee agreed that it would not have been possible to measure a true placebo effect if patients were made aware that both treatments were saline (not a bronchodilator) and one of the nebulizers was deliberately made to run inefficiently.

Patients were recruited on the Respiratory Wards of a University hospital. We recruited patients who had diagnosis of COPD confirmed by a respiratory consultant (patients with asthma or bronchiectasis were excluded from the study). Patients were approached by one of the investigators whilst in a relatively stable phase prior to discharge from hospital following an admission for exacerbated COPD. All testing was undertaken between 12.00 and 16.00, at least four hours after bronchodilator treatment.

Prior to participating in the study, patients gave informed consent and undertook baseline measurement of FEV1 and FVC using the best of 3 blows on a Microlab 3300 Spirometer (Micro-Medical LTD, Rochester, UK. Peak Expiratory Flow (PEF) was measured using a Wright's Peak Flow meter. Each patient also recorded an assessment of their perceived level of breathlessness using a seven point modified Likert scale (1 = Not breathless, 2 = Very mild breathlessness, 3 = Mild breathlessness, 4 = Moderate breathlessness, 5 = Severe breathlessness, 6 = Very severe breathlessness, 7 = Worst possible breathlessness).

Ten minutes after completion of nebulized therapy, FEV1, FVC and PEF measurements and subjective breathlessness scores were repeated. Patients also recorded a subjective assessment of benefit using the following modified Likert scale. (1 = No benefit from this treatment, 2 = Very slight benefit, 3 = Slight benefit, 4 = Moderate benefit, 5 = Good benefit,, 6 = Very good benefit, 7 = Best possible benefit).

Patients then received 4 puffs of salbutamol (400 mcg) using a Metered Dose Inhaler and 750 ml Volumatic spacer (Glaxo Smith Kleine UK).

Fifteen minutes later, FEV1, FVC and PFR measurements and subjective breathlessness scores and symptom relief scores were repeated.

All data was entered on a SPSS version 9 statistical package. Mann Whitney U-test was used to compare lung function tests and symptom relief scores. Wilcoxon Signed Rank test was used to compare the change in breathlessness scores for matched pairs before and after nebulized saline.

The study was approved by Salford and Trafford Research Ethics Committee. All patients gave written informed consent to partake in the study and to receive a single dose of nebulized treatment (in addition to all usual treatment).

## Results

34 patients completed the study; patient details are summarised in table 1. 6 patients took part in the study twice (once in each limb). This allowed 20 treatments with each nebulizer system to be compared.

The baseline FEV1 of the two treatment groups was well matched. Both groups had a non-significant fall in FEV1 after nebulized saline therapy and a small rise in FEV1 after 400 mcg salbutamol from MDI-spacer (Table 2). FVC, and PEF changes (not shown in table) were similar to FEV1 changes.

**Table 2:** Results All results expressed as Medians in top line and Mean (and 95% CI) in second line.

	**Active group**	**Placebo group**	**P value (*Mann Whitney*)**
**FEV1**	0.77	0.80	0.84
**Pre-treatment**	0.77 (0.65–0.89)	0.78 (0.61–0.95)	

**FEV1**	0.75	0.73	0.74
**Post nebulized saline**	0.73 (0.62–0.84)	0.75 (0.59–0.91)	

**FEV1**	0.80	0.77	0.63
**Post salbutamol MDI**	0.81 (0.67–0.94)	0.79 (0.62–0.96)	

**Breathlessness**	4	4	0.34
**Score 1 (Pre-treatment).**	3.9 (3.6–4.3) (*1 = Not breathless, 7 = Worst possible breathlessness*)	3.5 (3.0–4.0)	

**Breathlessness**	3	4	
**Score 2 (Post nebulized saline)**	3.0 (2.6–3.5)	3.3 (2.8–3.9)	0.85
**Wilcoxon test Score 1 V Score 2**	**<0.0001**	0.37	

**Breathlessness**	3	3	0.35
**Score 3 (Post salbutamol MDI)**	2.9 (2.5–3.3)	3.0 (2.6–3.5)	
**Wilcoxon test Score 2 V Score 3**	0.43	**0.014**	


**Symptom Relief**	3	1	**0.0002**
**Score Post nebulized saline**	3.1 (2.7–3.6) (*1 = No benefit, 7 = Best possible benefit*)	1.7 (1.2–2.3)	

**Symptom Relief**	3	3	0.37
**Score Post salbutamol MDI**	3.2 (2.7–3.7)	2.9 (2.4–3.4)	

The placebo group had a 4% improvement in breathlessness after treatment (Wilcoxon p = 0.37) compared with a 23% improvement after active nebulized saline (Wilcoxon p = 0.0001). This corresponded to a reduction from 4/7 (moderate breathlessness) before treatment to 3/7 (mild breathlessness) after treatment in the active treatment group.

The mean symptom relief score (patient's assessment of benefit) for the active treatment was 3/7, (slight benefit) almost identical to the response to 400 mcg salbutamol from MDI. The placebo group had a score of 2/7(very slight benefit) after nebulized placebo and 3/7(Slight benefit) after 400 mcg salbutamol from MDI.

15 patients in the active group felt better after nebulized saline, 5 felt the same and no patient felt worse. Six patients in the placebo group felt better after nebulized treatment, 12 felt the same and 2 felt worse (Chi Squared test p = 0.013).

Patients were asked if the nebulized treatments had any effect other than relief of breathlessness. 13 patients in the active group (65%) said that the nebulized treatment assisted sputum expectoration. Only 1 patient in the placebo group reported this effect (Difference between groups: -Fisher exact test, p = 0.0001). No patient reported any adverse effects from either nebulized treatment.

## Discussion

This is the first study which has compared active nebulized saline with placebo nebulized saline. The results suggest that nebulized saline has non-bronchodilator therapeutic effects that are possibly explained by airway-moistening and sputum-inducing effects of nebulized saline. Sputum volume was not measured in the present study but two thirds of patients who were given nebulized saline through an efficient nebulizer system reported that it helped them to expectorate sputum. This finding is consistent with the results of previous studies which have shown improved sputum clearance and decreased breathlessness following the open administration of nebulized saline [1,3] The results of the present study may be explained by airway-moistening and sputum-inducing effects of nebulized saline, both isotonic and hypertonic [1-9] The study of Vlachos-Mayer and colleagues [5] showed that most asthmatic patients required hypertonic saline to achieve sputum induction but more than half of COPD patients achieved sputum induction with nebulized isotonic saline (similar to the finding that 65% of COPD patients in the present study reported enhanced sputum clearance following nebulized saline).

Previous studies have shown that nebulized saline can have a bronchoconstrictor effect in some patients which is greater with hypertonic saline than with isotonic saline and greater in asthma patients than COPD patients [5,6,8,9] Nebulized isotonic saline had no significant effect on FEV1 in the study of Poole et al [3] or in the present study.

The main strength of the present study is inclusion of a placebo limb using an inefficient nebulizer system. Patients in the placebo group believed that they were receiving a nebulized treatment because a placebo effect could have been abolished if patients were told that both treatments involved no active medication and one of the patients involved an inefficient nebulizer system. This issue was discussed fully with the ethics committee and found to be acceptable because the patients did not miss any of their regular medication and they did not receive any pharmacological treatment. The use of a mouthpiece ensured that patients could not see or feel that the output from the experimental system was different to the nebulized bronchodilator therapy which they had received during their hospital admission (usually via a facemask). Furthermore, only 6 patients took part in both limbs of the study so most patients could not have tried to guess which treatment was more effective.

The 23% improvement in breathlessness in the active group was equivalent to the subjective benefit following 400 mcg of salbutamol from MDI-spacer. This improvement in breathlessness occurred without bronchodilation, mirroring the findings of Poole et al [3]. Based on the patients' observations and the results of previous studies, we believe that the therapeutic effect of nebulized saline may be produced by enhanced sputum clearance. A previous study at this hospital showed a similar subjective response to nebulized saline (given at 7 am) but the previous study also reported an improvement in FEV1 and PEF [13]. Patients in the previous study received nebulized saline on awakening, prior to their first bronchodilator treatment of he day. In these circumstances, it is likely that the nebulized saline assisted the expectoration of copious overnight secretions in the airways with some subsequent improvement in airflow. Patients in the present study were treated at about mid-day, having had bronchodilator therapy on awakening. It is therefore not surprising that the beneficial effects of nebulized saline were more modest in the present study. However, this study lends support to the common clinical practice of allowing patients with COPD to have nebulized saline "as required" as a supplement to regular nebulized bronchodilator therapy. This may assists sputum expectoration and relieve breathlessness without the side-effects that would occur if additional beta agonist treatment were given.

This study is in agreement with previous studies which have shown no bronchodilator effect (or a small bronchoconstrictor effect) when nebulized saline is given to patients with COPD [3]. This justifies the continuing use of nebulized saline as a placebo treatment in clinical trials of bronchodilator therapy which measure rise in FEV1 or PEF as the primary outcome measure.

However, as nebulized saline has non-bronchodilator therapeutic effects, it cannot be used as an inert placebo treatment in clinical studies where breathlessness or quality of life are to be measured. For example, Jenkins et al concluded that nebulized treatment had a strong placebo effect because patients expressed a preference for nebulized treatment even though the same bronchodilator effect could be achieved for most of their patients when nebulized saline was given with active bronchodilator therapy from a MDI device [10]. It is likely that many of these patients experienced a non-bronchodilator therapeutic benefit such as enhanced mucus clearance during nebulized saline therapy. It would be possible to co-administer nebulized saline with MDI bronchodilator therapy as an alternative to nebulized bronchodilator therapy for some patients with COPD who report difficulties with mucus clearance. However, this would be more inconvenient than nebulized bronchodilator therapy (and at least as expensive). For future clinical trials it would be possible to have two control groups, one receiving nebulized saline using an efficient system and one group using an inefficient system such as that used in the present study. This would allow investigators to assess whether nebulized saline had any therapeutic effect on their patients and it would also assess the true placebo response rate.

British and European nebulizer guidelines state that most patients with airflow obstruction should be treated with hand-held devices unless they have demonstrated clear additional benefit from the use of nebulized treatment in carefully monitored domiciliary studies [12,14]. The present study supports these recommendations, especially the provision that some patients may be commenced on nebulized treatment on the basis of substantial subjective benefit even if an additional bronchodilator response cannot be demonstrated. This study also supports the present practice of many physiotherapists and doctors who use nebulized isotonic saline to assist sputum clearance for patients with COPD who have difficulty in expectorating sputum.

## Abbreviations

FEV1 Forced Expiratory Volume in 1 second

FVC Forced Vital Capacity

PEF Peak Expiratory Flow

COPD Chronic Obstructive Pulmonary Disease

MDI Metered dose inhaler

## Competing interests

The authors declare that they have no competing interests.

## Authors' contributions

BROD developed the concept for the study and both authors designed the study protocol.

SYK recruited patients and performed all study measurements.

Both authors assisted in analysis of the data and preparation of the manuscript.

## Pre-publication history

The pre-publication history for this paper can be accessed here:


